# Three-dimensional microCT imaging of mouse development from early post-implantation to early postnatal stages

**DOI:** 10.1016/j.ydbio.2016.09.011

**Published:** 2016-09-23

**Authors:** Chih-Wei Hsu, Leeyean Wong, Tara L. Rasmussen, Sowmya Kalaga, Melissa L. McElwee, Lance C. Keith, Ritu Bohat, John R. Seavitt, Arthur L. Beaudet, Mary E. Dickinson

**Affiliations:** a Department of Molecular Physiology and Biophysics, Baylor College of Medicine, Houston, TX 77030, USA; b Optical Imaging and Vital Microscopy Core, Baylor College of Medicine, Houston, TX 77030, USA; c Department of Molecular and Human Genetics, Baylor College of Medicine, Houston, TX 77030, USA; d Cardiovascular Research Institute, Baylor College of Medicine, Houston, TX 77030, USA

**Keywords:** IMPC, MicroCT, Embryonic lethal screening

## Abstract

In this work, we report the use of iodine-contrast microCT to perform high-throughput 3D morphological analysis of mouse embryos and neonates between embryonic day 8.5 to postnatal day 3, with high spatial resolution up to 3 μm/voxel. We show that mouse embryos at early stages can be imaged either within extra embryonic tissues such as the yolk sac or the decidua without physically disturbing the embryos. This method enables a full, undisturbed analysis of embryo turning, allantois development, vitelline vessels remodeling, yolk sac and early placenta development, which provides increased insights into early embryonic lethality in mutant lines. Moreover, these methods are inexpensive, simple to learn and do not require substantial processing time, making them ideal for high throughput analysis of mouse mutants with embryonic and early postnatal lethality.

## 1. Introduction

The mouse is an ideal model system for studying gene function and modeling human disease because of the high homology between mouse and human genome. The analysis of individual mouse mutants has provided significant insights into the function of many mouse genes and families of genes. In addition, large-scale forward and reverse genetic screens provide an unbiased, systematic analysis of gene function and these efforts have revealed many novel genes with important roles. As a member of the International Mouse Phenotyping Consortium (IMPC), our group and others are engaged in the systematic phenotype analysis of mice carrying null alleles in the approximately 20,000 predicted protein encoded mouse genes (Adams et al., 2013; Dickinson et al., 2016). Homozygous null mutations that produce viable offspring are characterized using an adult phenotyping pipeline with tests aimed at identifying defects affecting behavior, movement, skeletal morphology, cardiac function, hearing, eye morphology, fertility, immunological function, hematopoiesis, etc. However, about 30–35% of the mouse genome has been estimated to encode genes that are essential for embryonic and early postnatal development (Adams et al., 2013; Dickinson et al., 2016). Homozygous recessive mutations with embryonic and postnatal lethality (zero homozygous null pups recovered at postnatal day 14) or subviability (less than 12.5% homozygous null pups recovered at postnatal day 14) provide an essential resource for those studying embryonic development and the mechanisms of congenital birth defects. Several phenotyping centers within the IMPC, including ours, have established high-throughput phenotyping pipelines for recessive lethals based on considerable input from the mouse genetics and development community (Dickinson et al., 2016). The goal of the embryonic lethal phenotyping pipeline is to establish a window of lethality during embryogenesis, to describe gross anatomical defects, and to characterize structural defects within the embryo using 3D volumetric imaging methods. These methods include high resolution episcopic microscopy (HREM), optical projection tomography (OPT), and micro computed tomography (microCT), All these methods have their strengths and limitations when applied for embryo phenotyping (Metscher, 2009a; Mohun and Weninger, 2012; Sharpe et al., 2002; Weninger et al., 2006; Wong et al., 2013b, 2015a).

HREM is a method that combines histology and 3D imaging and is one of the highest resolution methods to image entire 3D volumes of mouse embryos at any stage (Mohun and Weninger, 2012; Weninger et al., 2006). High-resolution images are collected from the block face of an embedded embryo followed by microtome section, repeated imaging and 3D image reconstruction. Although this provides very high-resolution 3D volume data and can be used with embryos of any stage or virtually any tissue sample, widespread use is limited because the systems are not commercially available, embedding and sectioning are time consuming, and increasing throughput requires multiple systems running simultaneously.

OPT was first described by James Sharpe's group (Sharpe et al., 2002) and is a method that images backscattered fluorescence from a transparent sample that is rotated around a central axis. Images are reconstructed into a 3D volume and light scatter is minimized by sufficient optical clearing. 3D volumes with relatively high spatial resolution (5–15 μm) can be obtained from samples that are several millimeters. Image volumes of whole mouse embryos from embryonic day 9.5 (E9.5) to E12.5 can be generated with fairly rapid acquisition, using either autofluorescence or specific fluorescent labels for contrast. Because of these advantages, OPT was identified as an appropriate method for producing images from E9.5 or E12.5 mutant embryos (Dickinson et al., 2016; Sharpe et al., 2002; Wong et al., 2015b). Embryos larger than this become increasingly difficult to clear and demand a large depth of field, which results in reducing the spatial resolution that can be obtained. Although a serious impediment to the popularization of this method has been the lack of a commercially available, off-the-shelf system, a recent publication has described intricate and robust plans for building a relatively inexpensive OPT system (Wong et al., 2013a).

MicroCT is another exciting 3D imaging tool for fixed specimens and several papers have shown beautiful, high-contrast, high-spatial resolution data from E15.5 mouse embryos enabling robust 3D segmentation and analysis of developing tissues and organs (Degenhardt et al., 2010; Metscher, 2009a, 2009b; Wong et al., 2012). To increase the contrast from the soft tissues, mouse embryos are soaked in iodine solution (Wong et al., 2012) and advances such as hydrogel stabilization (STABILITY) (Wong et al., 2013b) helps to prevent shrinkage of samples in the contrast solutions. Unlike HREM and OPT, bench-top microCT units can be purchased from several manufacturers. These units are capable of imaging with high spatial resolution (as high as 1–3 μm) and image acquisition can be automated together with multi-sample holders to support high throughput analysis.

Given the advantages of microCT imaging (commercial availability, high spatial resolution, contrast enhancement, multi-sample handling), we sought to determine whether microCT could be extended for use from early post-implantation to early postnatal stages. The goal was to determine whether microCT could be used as a one-instrument-fits-all, commercially available solution for embryonic phenotype analysis that any group interested in embryonic analysis could implement. Here we show that microCT can be used for 3D imaging from E8.5 to postnatal day 3 (P3), producing high-resolution datasets that can be used to detect phenotype abnormalities. Although we were initially skeptical that sufficient contrast could be achieved given the thin, soft tissues of early stage embryos, we were pleased to obtain high-contrast images that could readily be used to determine anatomical differences between wild type and mutant embryos. Finally, we determined whether microCT could be used to image early post-implantation embryo within the yolk sac and the optically opaque decidua. We found that this approach was ideal for properly scoring anatomical features of the embryo and extra-embryonic structures, as well as embryo turning without disturbing the embryo during dissection. We also show that embryos can be processed for genotyping after 3D microCT image acquisition, demonstrating that aberrant phenotypes can be directly related to the mutant genotype. Overall, these studies represent an advance in the current approaches for the high-throughput analysis of embryonic phenotypes and will also benefit other researchers who are interested in studying structural changes during early post-implantation to early postnatal development in the mouse and potentially other species.

## 2. Results

### 2.1. MicroCT imaging of mouse embryos from embryonic day 9.5 (E9.5) to postnatal day 3 (P3)

**Fig. 1:**
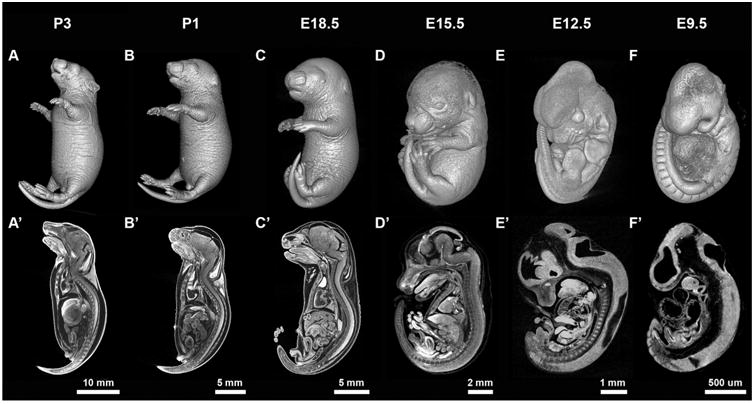
Early postnatal to early post-implantation mouse samples imaged by iodine contrast microCT. (A–F) Surface rendering and (A′–F′) a sagittal cross-section of mouse postnatal at P3 to embryonic at E9.5 samples generated by iodine contrast 3D volumes acquired on microCT.

To determine the range of embryonic and postnatal stages that could be imaged with microCT, we adapted existing microCT protocols at different stages between E9.5 and P3. For stages from E15.5 to P3, we have implemented the STABILITY protocol, which has been described previously by Wong et al. (Wong et al., 2013b) to generate a tissue-hydrogel complex to prevent sample deformation and organ shrinkage. Briefly, embryos and neonates were fixed by immersion in 4% paraformaldehyde, treated according to the STABILITY protocol and then were immersed in 0.1 N iodine solution. It was necessary to extend the time period of fixation and the incubation time in the iodine solution to allow for sufficient distribution of these reagents through the embryos and neonates (Table 1). Fig. 1 shows a surface rendering (Fig. 1A–D) and a sagittal cross-section (Fig. 1A′–D′) from imaged 3D volumes of P3 to E15.5 acquired with a 0.5 mm aluminum attenuation filter at 11 μm voxel size (Supplementary Movies 1–4). We assessed the 3D image data acquired using these parameters and found even contrast throughout similar tissues with sharp boundaries at tissue borders. Shorter iodine staining time resulted in insufficient contrast to resolve details for the organs at center, such as heart, lung, liver, and intestines, especially for early postnatal stage samples (data not shown). However, it is relatively easy to check whether the sample is ready for imaging by simply loading the sample on to the scanner and see if the center region can be well resolved by taking a single shadow projection image before proceeding for full data acquisition. Despite the use of the STABILITY protocol, there was still some evidence of shrinkage in P1 and P3 embryos as indicated by the separation between the brain and the skull cavity, but overall we found that this method produced 3D volume data with clear histology-like definition of organ and tissue structures, necessary for identification of anatomical features, as well as shorten the time required for iodine staining to generate sufficient contrast (e.g. 2 weeks for non-stabilized E18.5 samples compared to 3–5 days after stabilization).

Supplementary material related to this article can be found online at http://dx.doi.org/10.1016/j.ydbio.2016.09.011.

Next, we focused on determining whether iodine-contrast microCT imaging can be applied for acquiring high-resolution 3D volume images of E12.5 and E9.5 mouse embryos. For these stages, embryos were fixed by immersion in 4% paraformaldehyde, stained in 0.1% iodine solution overnight, and then imaged on microCT (Table 1). Because of the small size and limited tissue density in these early post-implantation embryos, iodine in the stained sample diffuses out into the mounting agarose rapidly. Because of this we observed a reduction in image contrast during image acquisition. To overcome this issue, we optimized the protocol to limit the volume of mounting agarose to reduce the speed and amount of iodine diffusion, as well as shorten the data acquisition time. We have optimized the protocol to image with a 5 μm voxel size for E12.5 and a 3 μm voxel size for E9.5 embryos to sufficiently resolve the anatomical structures at each individual stage. The data acquisition times were also limited to 75 min and 50 min after mounting, respectively. We have also tested and selected to use no attenuation filter to capture the full dynamic range of iodine-generated contrast at these two stages. Using this optimized protocol, 3D microCT volumes of early post-implantation embryos revealed high contrast data with clear anatomical features (Supplementary Movies 5 and 6), i.e. first and second branchial arches, heart tube, as well as somites, without the requirement of further post-processing of the reconstructed data (Fig. 1E and F). Sagittal views of both E12.5 and E9.5 embryos also showed that the 3D data acquired on microCT can be used to resolve anatomical features within embryos (Fig. 1E′ and F′).

Supplementary material related to this article can be found online at http://dx.doi.org/10.1016/j.ydbio.2016.09.011.

**Fig. 2:**
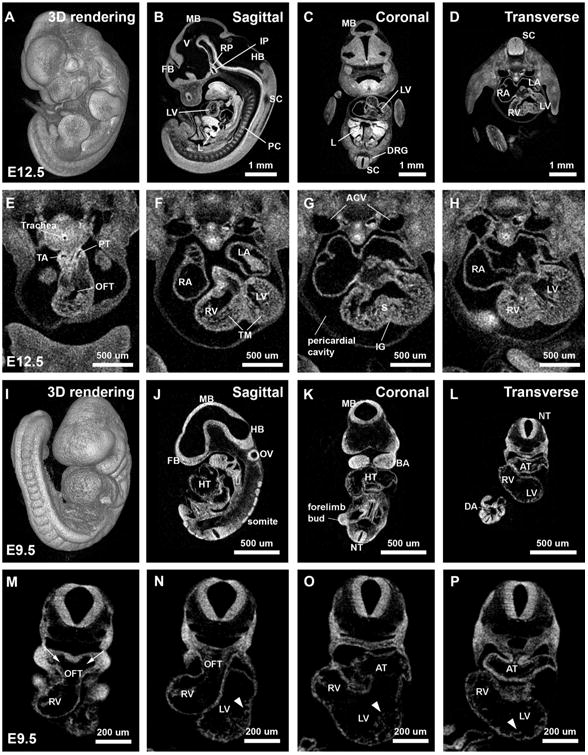
High resolution 2D virtual sectioning of reconstructed E12.5 and E9.5 microCT datasets through different body planes and through the developing heart. Surface rendering and digital sections of the 3D volumes from sagittal, coronal, and transverse planes of both (A – D) E12.5 and (I – L) E9.5 embryos, as well as transverse sections through the heart at four levels from the anterior to the posterior on both (E – H) E12.5 and (M – P) E9.5 embryos. The high spatial resolution and clear contrast images make is possible to clearly define anatomical features for both E12.5 and E9.5 stages. V: ventricles of the brain. FB: forebrain. MB: midbrain. HB: hindbrain. IP: infundibulum of pituitary. RP: Rathke's pouch. PC: primordial cartilage of the vertebrae. L: liver. SC: spinal cord. DRG: dorsal root ganglion. RA: right atrium. LA: left atrium. RV: right ventricle. LV: left ventricle. TA: thoracic aorta. PT: pulmonary trunk. OFT: outflow tract. TM: trabeculated myocardium. ACV: anterior cardinal veins. S: interventricular septum. PC: pericardial cavity. IG: interventricular groove. HT: heart tube. OV: otic vesicle. BA: first branchial arches. NT: neural tube. DA: dorsal aorta. AT: common atrium. Arrowhead: trabeculae.

To further examine internal anatomical details, we examined digital sections (sagittal, coronal, and transverse planes) extracted from a full 3D volume of a E12.5 (Fig. 2A–D) and a E9.5 (Fig. 2I–L, and the high resolution digital z-stack sections through the transverse plane is available for use with the Virtual Microscopy as eSlide: VM03103) embryo, as well as transverse sections through the heart at four levels from both stages (Fig. 2E–H, M–P). The high spatial resolution and clear contrast make it possible to clearly define anatomical features, such as ventricles of the brain (V), forebrain/midbrain/hindbrain (FB, MB, HB), infundibulum of pituitary (IP), Rathke's pouch (RP), primordial cartilage (PC) of vertebrae, liver (L), dorsal root ganglion (DRG), right and left ventricles (RV, LV) and atria (RA, LA) in E12.5 embryos (Fig. 2B–D), as well as somites, otic vesicle (OV), first branchial arches (BA), heart tube (HT), neural tube (NT), and dorsal aorta (DA) in E9.5 embryos (Fig. 2J–L). Digital sections through the developing heart, at E12.5 (Fig. 2E–H), clearly show that the spongy meshwork of the trabeculated myocardium (TM) within the right and left ventricles (RV, LV) has begun to compact and the interventricular septum (S) is evident dividing the LV from the RV. Other anatomical features, including the thoracic aorta (TA), pulmonary trunk (PT), the left and right anterior cardinal veins (ACV), and the pericardial cavity surrounding the heart are also visible. Images from the E9.5 microCT volume (Fig. 2M–P) also show that the outflow tract (OFT), the future RV and LV, and the common atrium (AT) are clearly discernible. At this stage, trabeculae (Fig. 2N–P, arrow head) are just forming and are clearly visible as finger-like projections in the ventricles, most obviously in the LV. Together, these data show that microCT can be used to produce high-quality image data for observing embryo and early postnatal anatomy from a wide range of developmental stages using simple iodine contrast.

The STABILITY protocol was not performed for these stages because the manual removal of excess hydrogel is technically challenging with such small, fragile samples. Although even contrast was obtained without STABILITY, we did observe some shrinkage of these early post-implantation samples due to dehydration. However, the shrinkage did not impair our ability to observe internal anatomic features as shown in Fig. 1E′ and F′, but variations in shrinkage could certainly impair our ability to determine differences in mutant size from microCT volumes and should be determined before embryo processing.

### 2.2. MicroCT imaging of mouse embryo within yolk sac and decidua

**Fig. 3:**
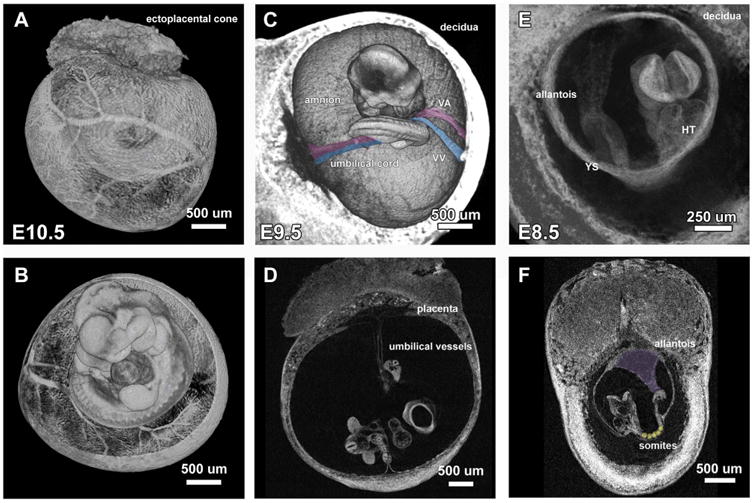
MicroCT imaging of E10.5 to E8.5 mouse embryos within extra-embryonic structures. Iodine contrast E10.5 embryo within yolk sac imaged on microCT revealed (A) the remodeled vasculature on the yolk sac and (B) the orientation of the embryo within the yolk sac. E9.5 embryo imaged within decidua shows (C) the connection of vitelline vein/artery (pseudo-colored in blue/pink) and umbilical vein/artery (pseudo-colored in pink/blue) to yolk sac and placenta and (D) 2D virtual section of the E9.5 embryo within decidua reveals the diameter and length of remodeled umbilical vessels. (E) E8.5 embryo imaged within decidua shows the original orientation of the unturned embryo with the allantois extending from the tail toward the chorion. (F) 2D virtual section of an E8.5 embryo shows the sharp boundary of the somites (pseudo-colored in yellow) and how the allantois extends to the chorion (pseudo-colored in purple).

As demonstrated previously, iodine-contrast microCT imaging can be used to generate 3D volumes of mouse embryos as early as E9.5. Given the ability of microCT to image thick samples, we tested whether microCT could be used to image embryo within the yolk sac and decidua between the stages of E8.5 to E10.5. Embryos were dissected from the uterus but were kept within the yolk sac, fixed in 4% paraformaldehyde, stained in 0.1 N iodine solution overnight, and then imaged at 3 μm voxel size with no attenuation filter selected. As described above, embryos within the yolk sac were mounted in limited amount of agarose and the imaging time was also limited to prevent loss of contrast during data acquisition. Fig. 3A and B show the result of an E10.5 embryo imaged within yolk sac with chorioallantoic placenta still attached (Supplementary Movie 7, and the 3D rendered movie is also available for use with the Virtual Microscopy as eSlide: VM03104). The remodeled vasculature within the yolk sac can be well resolved by iodine contrast microCT imaging without specific antibody labeling as the blood cells readily absorb the contrast and remain distributed in the vessels of rapidly fixed embryos (Fig. 3A). In addition, the orientation of the embryo was also preserved within the yolk sac (Fig. 3B). E9.5 embryos can also be imaged within yolk sac using the same approach (data not shown). For both E10.5 and E9.5 embryos, the yolk sac remained inflated and intact after the iodine staining in the majority of embryos processed (n=54/79). For those yolk sacs that were deflated, there was clear evidence of that the yolk sac was punctured or torn in all cases, suggesting that damage during dissection and handling caused deflation rather than the iodine staining procedure.

Supplementary material related to this article can be found online at http://dx.doi.org/10.1016/j.ydbio.2016.09.011.

Next, we tested the possibility of imaging E9.5 and E8.5 embryos within the deciduum, to fully preserve embryo to extra-embryonic associations and embryo orientation. Fig. 3C and D shows that E9.5 embryos can be imaged within the decidua without disturbing the orientation or morphology (Supplementary Movie 8). Fig. 3C shows the vitelline vein/artery (pseudo-colored in blue/pink) and umbilical vein/artery (pseudo-colored in pink/blue) connecting the yolk sac and placenta. In fact, anatomical details as thin as the amnion surrounding the embryo can also be resolved. 2D virtual sections of the data also reveal the diameter and length of the remodeled umbilical vessels, as well as vitelline artery and vein, which can be measured to provide quantitative data related to embryonic-extra embryonic circulation (Fig. 3D). Fig. 3E and F show an E8.5 embryo imaged within the decidua (Supplementary Movie 9). The embryo within the yolk sac (Fig. 3E, YS) is clearly visible, as are details of the heart tube (HT), developing allantois and the anterior neural folds. A 2D virtual section through the volume shows the sharp boundary of the somites (pseudo-colored in yellow) and the clear connection between the allantois and the chorion (pseudo-colored in purple) (Fig. 3F), which makes it possible to stage the embryos using somite counts and to identify early defects in embryo-placenta formation. Moreover, unlike imaging the embryo alone or within the yolk sac, the dense decidua limits iodine diffusion out to the mounting agarose and we found that the iodine contrast tissue remains sharp even days after mounting. Thus, it is possible to image the whole litter by mounting multiple deciduum within a single sample tube and then scanning each one sequentially for high throughput screening.

Supplementary material related to this article can be found online at http://dx.doi.org/10.1016/j.ydbio.2016.09.011.

### 2.3. MicroCT imaging of Rad9a E9.5 null embryos

**Fig. 4:**
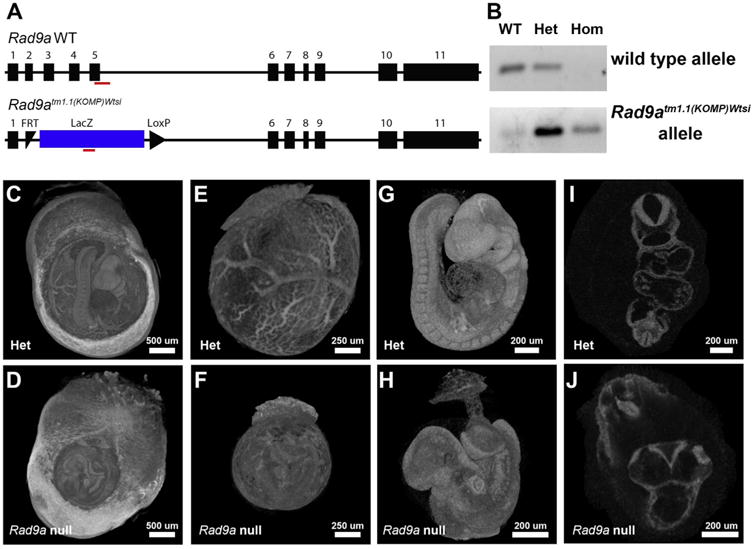
*Rad9a* knockout mouse is embryonic lethal with severe developmental defects at E9.5. (A) Schematic diagram of the wild type and the knockout alleles of *Rad9a*. Red line under each allele indicates the amplicon region used for genotyping primer design. (B) PCR result of the iodine contrast sample to confirm genotypes. (C and D) The entire litter from a heterozygote × heterozygote *Rad9a^tm1.1(KOMP)Wtsi^* cross were imaged within deciduum. (E and F) Embryos within yolk sacs and (G and H) by themselves along with the umbilical cord connecting to the placenta were digitally segmented out from the original data set. (E and F) *Rad9a^tm1.1(KOMP)Wtsi^* null embryo is smaller in size with un-remodeled yolk sac vasculature compared to heterozygous littermate. (G and H) At E9.5, *Rad9a* null embryo did not turn, the anterior neural folds were still open, the ventral midgut region remained open, and the posterior part of the null embryo is severely dysmorphic. (I and J) The heart tube in *Rad9a^tm1.1(KOMP)Wtsi^* remained linear without undergoing further looping process compared to the left/right atrium and ventricles formed in heterozygote.

Next, we wanted to determine if we could image mutant embryos within deciduum with microCT and then recover genotype information using PCR. For these studies, we examined embryos from heterozygote × heterozygote crosses using *Rad9a^tm1.1(KOMP)Wtsi^* mice. *Rad9a* encodes RAD9 checkpoint component clamp A (RAD9A). RAD9A associates with HUS1 and RAD1 to form 9-1-1 complex, which is known to regulate cell cycle checkpoints in mitosis and in response to DNA damage (Hopkins et al., 2004; Parrilla-Castellar et al., 2004). Deletion of *Rad9a* in mice has shown that homozygous E9.5 *Rad9a* null embryos were dysmorphic, failed to turn and had underdeveloped hearts and brains (Hopkins et al., 2004). The entire litter from a heterozygote × heterozygote *Rad9a^tm1.1(KOMP)Wtsi^* cross was harvested from the uterus of the pregnant dam but the embryos were kept within the decidua. These samples were processed for iodine-contrast microCT as discussed above, but then following the imaging, each embryo within decidua was washed with 10% (w/v) sodium thiosulfate to reduce iodine (I_2_) to water-soluble iodide (I^-^) followed by extensive washes. Genomic DNA was then extracted from embryos and used for genotyping. Fig. 4A shows the schematic diagram of the wild type and the knockout alleles of *Rad9a*. The red line under each allele indicates the amplicon region that was used for designing the primer sets for genotyping (detailed information of the targeting vector design and knockout strategy can be found on IMPC website http://www.mousephenotype.org). Fig. 4B shows the PCR result from DNA isolated from embryos imaged with iodine-contrast microCT, revealing the genotypes. Fig. 4C–J show image data from the confirmed heterozygote and *Rad9a^tm1.1(KOMP)Wtsi^* null embryos. Although wild type and heterozygous embryos were morphologically indistinguishable, *Rad9a^tm1.1(KOMP)Wtsi^* null embryo (Fig. 4D and F) was much smaller than the heterozygote (Fig. 4C and E) and the yolk sac vasculature was an un-remodeled, primitive plexus (Fig. 4F) compared to the remodeled vessel hierarchies evident in the heterozygous embryo (Fig. 4E). Close examination of imaged volumes from *Rad9a* null embryos indicated that the allantois was connected to the chorion but the embryos did not turn, the anterior neural folds were still open, the ventral midgut region remained open, and the posterior part of the null embryo is severely dysmorphic (Fig. 4G and H). Digital sections through the image volume also showed that while the left/right atrium and ventricles had formed in the heart tube of heterozygous embryos (Fig. 4I), the heart tube in *Rad9a^tm1.1(KOMP)Wtsi^* null embryo remained linear and did not undergo normal looping (Fig. 4J). These data show strong concordance with previously published phenotype information (Hopkins et al., 2004). These data show that phenotype analysis for early post-implantation embryos can be carried out in an undisturbed manner, followed by genotyping after 3D microCT image acquisition.

## 3. Discussion

In this study, we have demonstrated that commercially available microCT instrumentation and software can be easily implemented for 3D volume imaging with high spatial resolution to study mouse development from early post-implantation (E8.5) to early postnatal (P3) stages. While high-resolution 3D volume imaging with methods such as OPT and microCT cannot yet replace the cellular and sub-cellular level contrast provided by histological stains of 2D sections, these 3D methods do enable rapid, *in toto* analysis of structural abnormalities. Thus, they are a convenient means to identify embryonic phenotypes in mutant mouse lines and are particularly effective in high-throughput screens to identify and curate phenotype information. The major advance put forth here is that a single, commercially available method, iodine-contrast microCT, can be used to image embryos throughout embryogenesis and early postnatal stages. These data show that microCT can be an alternative to using different modalities at different stages and/or systems that require expertise to first build before they can be used, such as OPT. Using microCT we were also able to show that it is possible to image embryos without having to dissect them away from vital extra-embryonic tissues and acquire genotype information after imaging, which provides a new, efficient way to identify early defects in vessel remodeling, allantois formation and fusion, and the initial stages in placental development in mutants.

While we were able to implement the STABILITY protocol for late embryonic and early post-natal samples (E15.5-P3), isolated E9.5 and E12.5 embryos were too fragile for us to implement the STABILITY protocol without significant damage. Embryos imaged within deciduum preserve not only the orientation of the embryos but also reveal the vascular connections to the yolk sac and placenta allowing for the detection of turning and placentation abnormalities in mutant lines.

We also show here that genomic DNA purification and PCR genotyping is possible after the samples have been treated with iodine solutions and imaged on microCT. This is particularly important for the younger embryos processed within the deciduum where it is not possible to retain part of the embryo for genotyping before processing. However, we did notice that once the embryos were stained with iodine solution, genomic DNA was fragmented, based on electrophoretic gel mobility analysis and the presence of non-specific PCR amplifications (data not shown). Although it is still unclear how high concentration of iodine interferes with the integrity of the genomic DNA, we determined that if samples were processed in a timely manner and the amplicon size was smaller than 100 bp, consistent results were obtained for genotyping. However, improvements to this protocol are underway to reduce DNA fragmentation. For instance, because the early embryos are quite fragile, we have not yet determined if using the STABILITY protocol might help preserve DNA integrity, but this might be beneficial if STABILITY can be optimized for younger embryos.

In conclusion, we have shown that microCT is a commercially available instrument that can be used to characterize morphology and identify structural abnormalities in mutants from early post-implantation embryonic to early postnatal stages. Thus, a single tool can be used to obtain 3D volume images at multiple stages of embryogenesis and is suitable for high-throughput analysis such as for embryonic phenotyping pipelines. Furthermore, the development of a method to obtain high-resolution images of the undisturbed embryo within yolk sac and decidua in 3D, it opens the possibility for identifying potential defects in allantois, placenta, umbilical cord, and vitelline vessel remodeling using high-throughput analysis.

## 4. Materials and methods

### 4.1. Mice

All mouse lines in this study are derived from International Knockout Mouse Consortium (IKMC) ES cell resources. All mice are produced and maintained on a C57BL/6NJ genetic background. This study was carried out in strict accordance with the recommendations in the Guide for the Care and Use of Laboratory Animals of the National Institutes of Health. All animal research was conducted according to protocols approved by the Institutional Animal Care and Use Committee (IACUC) of Baylor College of Medicine (BCM).

### 4.2. Sample preparation and imaging parameters for microCT imaging

Embryos were dissected at different stages into warm PBS and were immediately fixed in 4% paraformaldehyde (Sigma) for different amounts of time dependent on their developmental stages (E8.5–E15.5: overnight at 4 °C; E18.5 to P3: 3 days at 4 °C. Details can also be found in Table 1). For embryos between E8.5 and E12.5, samples were washed with 1× PBS three times, and then immersed in 0.1 N (v/v) iodine solution (Sigma) overnight at room temperature. E8.5 to E10.5 samples were mounted individually in 400ul of 1% w/v agarose (AMRESCO) within a screw-cap microcentrifuge tube (2 ml, VWR) and imaged immediately. E12.5 samples were mounted in 3 ml of agarose in 5 ml tubes (VWR, 16 mm × 56 mm) and imaged immediately. For samples at E15.5 and beyond, STABILITY protocol was implemented to prevent sample shrinkage and deformation (Wong et al., 2013b) after fixation. The samples were transferred to 50 ml conical tubes and immersed in 20 ml of STABILITY buffer [4% w/v paraformaldehyde (pH 7.2, Sigma), 4% w/v acrylamide (Bio-Rad), 0.05% w/v bis-arcylamide (Bio-Rad), 0.25% w/v VA044 initiator (Wako Chemicals), 0.05% w/v Saponin (Sigma) in 1× PBS] and incubated at 4 °C for 3 days allowing polymer diffusion through the samples. The samples were then placed in a desiccator to replace the air in the sample tubes with nitrogen gas. The crosslinking reaction was initialized by incubating the samples at 37 °C for 3 h for the acrylamide-paraformaldehyde to crosslink with the tissue and created a hydrogel-tissue mixed structure. After crosslinking, the external hydrogels were removed from the specimens and the samples were stored in 1× PBS with 0.1% w/v sodium azide at 4 °C until ready for imaging. The staining time in iodine for stages between E15.5 to P3 was also listed in Table 1. For better contrast, the iodine solution was replaced every 3 days. Samples were then mounted in 56 mm capped sample tubes in 1% w/v agarose immediately before imaging.

The raw data for 3D imaging of the samples were acquired via SKYSCAN 1272 micro-CT scanner (Bruker). Each data set was acquired with the X-ray source at 70 kV and 142 μA with a 0.5 mm aluminum attenuation filter. For samples at E8.5 and E9.5, no filter was selected for image acquisition. The acquisition parameters were set to handle high throughput screening with high resolution images, where the acquisition time/isotropic voxel for each sample ranged between 50 min/3 voxel (E9.5) to 5 h/11 voxel (P3) depend on sample stages (Table 1). Each sample was rotated 180 degrees along the anterior-posterior (AP) axis and a projection image at 2016×1344 pixels was generated every 0.3° at an average of 3 images. Acquired projection images were then reconstructed by using Fledkamp Algorithm (Feldkamp et al., 1984) for cone-beam CT data via NRecon Reconstruction (Version: 1.6.9.8; Bruker) software.

### 4.3. Image processing

Reconstructed 3D data for each sample was rendered to view the whole volume in 3D via CTVox (Version: 2.6.0; Bruker), and it would be further processed for viewing the data set in different section planes. A custom software Harwell Automated Recon Processor (HARP, Version: 2.0.9; Harwell) was used to process each data set to crop and convert the image series into a single .nrrd file format (Brown et al., 2016). Once the files have been processed, all the data sets can be opened from a freeware Slicer (Version: 4.4.0; www.slicer.org) and viewed in coronal/sagittal/transverse section planes, as well as 3D volume rendering for detail morphological analysis of each sample. The segmentation of E9.5 heterozygote and *Rad9a^tm1.1(KOMP)Wtsi^* null embryos from the reconstructed images of the samples within the deciduum was done in CT-Analyser (CTAn, Version: 1.14.4.1; Bruker) by generating a series of ROI mask around the embryos to extract the information.

### 4.4. Iodine removal after image acquisition and genotyping

The production of *Rad9a^tm1.1(KOMP)Wtsi^* mouse line was done at BCM and detail information can be found on the IMPC website http://www.mousephenotype.org/data/genes/MGI:1328356. The entire litter from a heterozygote × heterozygote *Rad9a^tm1.1(KOMP)Wtsi^* cross was harvested from the uterus of the pregnant dam but the embryos were kept within the decidua and processed as described previously for imaging. After samples were imaged on microCT, E9.5 null *Rad9a^tm1.1(KOMP)Wtsi^* and littermates were removed from the sample tubes while still embedded in agarose gels and immersed in sterile Milli-Q water for an hour to rehydrate the samples. Samples were then washed in 10% w/v sodium thiosulfate in sterile Milli-Q water three times, one hour each to reduce the iodine (I^2^) to water soluble iodide (I^-^) and wash away from the samples. Samples were then washed with sterile Milli-Q water three times, one hour each. Samples were removed from the agarose gels and immersed in Milli-Q water overnight, and the embryos were then dissected out for genomic DNA isolation. Genomic DNA was purified by using the Gentra Puregene kit (Qiagen). PCR genotyping was performed with *Rad9a* loss-of-allele primers (forward: 5′ CCAGCCTCATGCCCTCACTTATTG 3′; reverse: 5′ AGGAGAGACTCAGGCGAAGTTTGAAG 3′) and lacZ primers (forward: 5′ CCCAGCGACCAGATGATCACAC 3′; reverse 5′ CGTAACGCGAATGGTGCAGC 3′) to detect wildtype and LacZ (mutant) alleles, respectively. PCR cycling conditions were 95 °C for 5 mins initially, followed by 34 cycles of 95 °C, 30 s; 62 °C, 30 s, 72 °C, 30 s, with a final extension at 72 °C for 5 mins.

## Supplementary Material

1

2

3

4

5

6

7

8

9

## Figures and Tables

**Table 1:** Sample preparation procedures and imaging parameters for microCT imaging from P3 to E9.5.

Stage	Fixation time	Stabilization	0.1 N iodine Staining	Resolution (voxel)	Attenuation filter	Acquisition time
P3	3 days	Yes	7–10 days	11 μm	0.5 mm Al	300 min
P1	3 days	Yes	7–10 days	11 μm	0.5 mm Al	225 min
E18.5	3 days	Yes	3–5 days	11 μm	0.5 mm Al	150 min
E15.5	Overnight	Yes	Overnight	11 μm	0.5 mm Al	75 min
E12.5	Overnight	Not performed	Overnight	5 μm	No filter	75 min
E9.5	Overnight	Not performed	Overnight	3 μm	No filter	50 min
